# Supplementary material for: Improving VAE based molecular representations for compound property prediction
Source: J Cheminform. 2022 Oct 14;14:69. doi: 10.1186/s13321-022-00648-x (PMC9569108; doi:10.1186/s13321-022-00648-x)
Supplement: Supplementary file 1 — Additional file 1: Table S1. Hyperparameters of CVAE (a) and PVAE (b) and size of the models (c) used for pre-training. Table S2. Hyperparameters of the 1D ResNet architecture. Table S3. Performance of various models for predicting downstream tasks (a) logS, (b) logD and (c) logBB. Fig S1. Correlation Heatmaps between descriptors calculated by RDkit package and aqueous solubility log S (a), lipophilicity logD (b) and blood-brain barrier penetration logBB (c) datasets. Fig. S2. Clusters of the molecules from logS dataset in the latent space of a PVAE jointly pre-trained with a MolLogP predictor. Clusters are obtained using K-Means algorithm with varying number of clusters. In all cases there is one outlier cluster, without which there is a correlation between RMSE error of logS prediction and KL divergence between the cluster and the prior distribution. [file 13321_2022_648_MOESM1_ESM.pdf]

Improving VAE based molecular representations for compound property prediction

Table S1 Hyperparameters of CVAE (a) and PVAE (b) and size of the models (c) used for pre-training.

(a)

Name	With property predictor	Without property predictor
PADDING	right	right
RAND_SEED	42	42
epochs	120	70
vae_annealer_start	29	29
dropout_rate_mid	0.082832929704794792	0.082832929704794792
anneal_sigmod_slope	0.51066543057913916	0.51066543057913916
recurrent_dim	488	488
hidden_dim	196	196
tgru_dropout	0.19617749608323892	0.19617749608323892
hg_growth_factor	1.2281884874932403	1.2281884874932403
middle_layer	1	1
prop_hidden_dim	67	-
batch_size	182	126
prop_pred_depth	3	-
lr	0.00045619868229310396	0.00039192162392520126
prop_pred_dropout	0.15694573998898703	-
prop_growth_factor	0.99028340731314179	-
momentum	0.99027641036225744	0.97170900638688007

(b)

Name	Value

batch_size	30
hidden_size	1024
embedding_size	30
epochs	72
rnn_type	gru
learning_rate	0.001
latent_size	196
n_layers	1
word_dropout	0.1
anneal_function	logistic
k0	2500
x0	0.0025

(c)

**Model**	**Num. parameters**
CVAE	4M
PVAE	7M

Fig S1. Correlation Heatmaps between descriptors calculated by RDkit package and aqueous solubility log S (a), lipophilicity logD (b) and blood-brain barrier penetration logBB (c) datasets

(a)

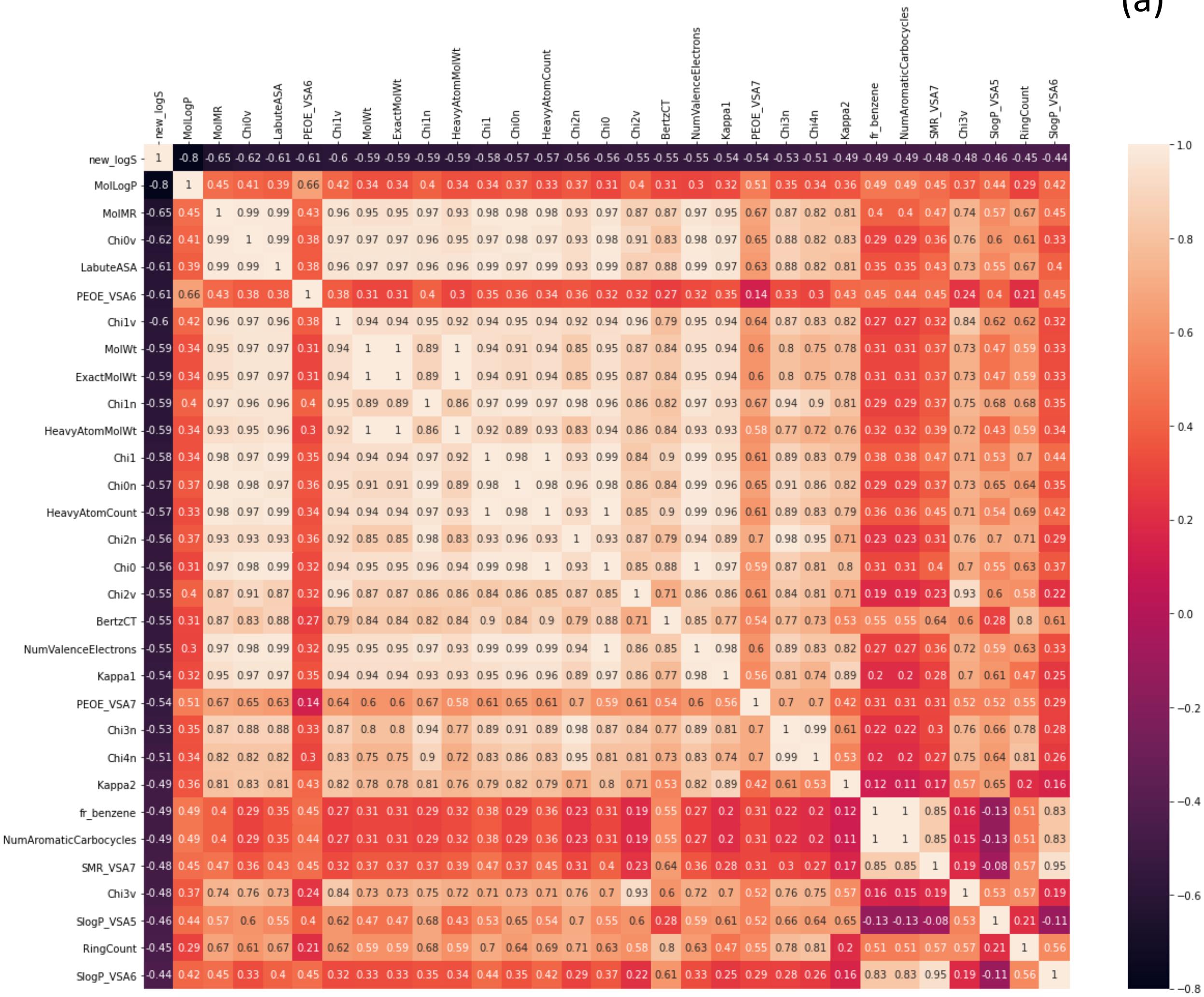

(b)

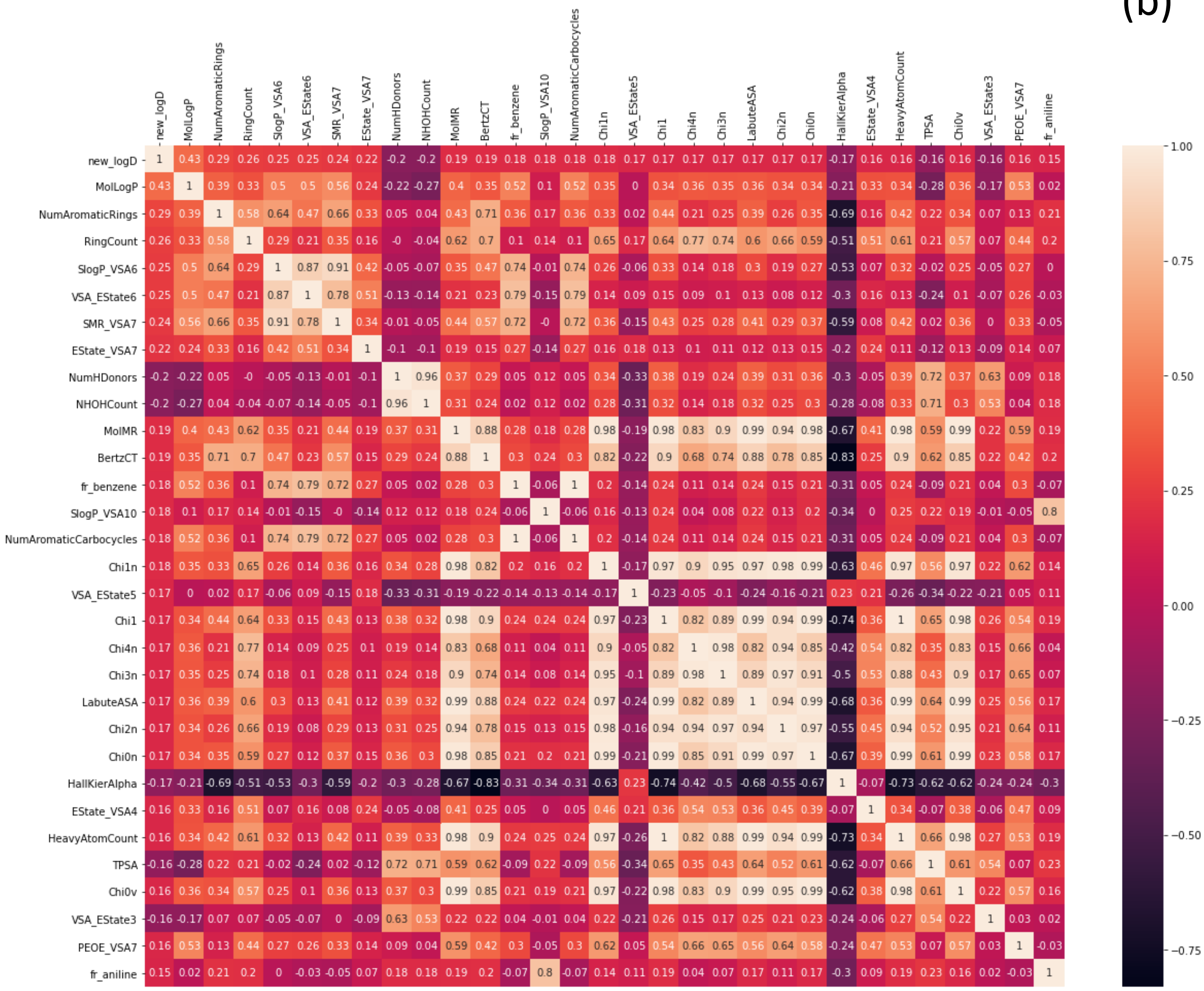
new_logD
MolLogP
NumAromaticRings
RingCount
SlogP_VSA6
VSA_EState6
SMR_VSA7
EState_VSA7
NumHDonors
NHOHCount
MolMR
BertzCT
fr_benzene
SlogP_VSA10
NumAromaticCarbocycles
Chi1n
VSA_EState5
Chi1
Chi4n
Chi3n
LabuteASA
Chi2n
Chi0n
HallKierAlpha
EState_VSA4
HeavyAtomCount
TPSA
Chi0v
VSA_EState3
PEOE_VSA7
fr_aniline
1.00
0.75
0.50
0.25
0.00
−0.25
−0.50
−0.75

(c)

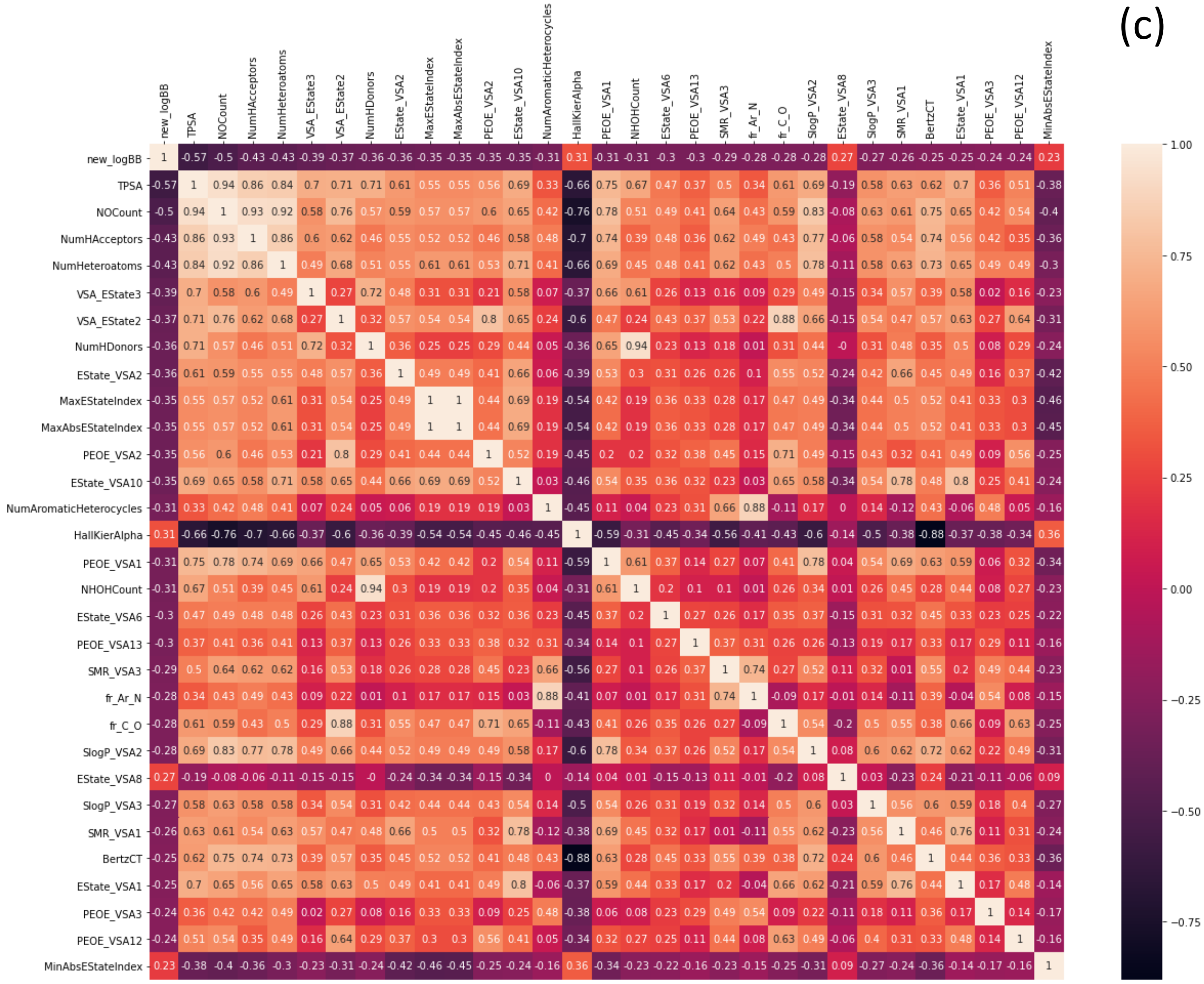
new_logBB
TPSA
NOCount
NumHAcceptors
NumHeteroatoms
VSA_EState3
VSA_EState2
NumHDonors
EState_VSA2
MaxEStateIndex
MaxAbsEStateIndex
PEOE_VSA2
EState_VSA10
NumAromaticHeterocycles
HallKierAlpha
PEOE_VSA1
NHOHCount
EState_VSA6
PEOE_VSA13
SMR_VSA3
fr_Ar_N
fr_C_O
SlogP_VSA2
EState_VSA8
SlogP_VSA3
SMR_VSA1
BertzCT
EState_VSA1
PEOE_VSA3
PEOE_VSA12
MinAbsEStateIndex
1.00
0.75
0.50
0.25
0.00
−0.25
−0.50
−0.75

Table S2 Hyperparameters of the 1D ResNet architecture

**Hyperparameter**	**Value**
Loss Function	RMSE (logS, logD), Binary cross entropy (logBB)
Size of convolution kernels	9
Number of filters of CNN	9
Strides	1
Number of layers of CNN	19
Number of fully-connected hidden layers	1
Number of neurons of fully-connected layer	970
Activation Function	ReLU
Optimizer	Adam
Learning Rate	10^{-5}
Weight initialization	Uniform
L2 Weight Decay	10^{-5}
Batch size	47
Epochs	2000 (logS), 1500 (logD), 500 (logBB)

Table S3 Performance of various models for predicting downstream tasks (a) logS, (b) logD and (c) logBB

(a)

	**1D ResNet** R^2**/RMSE**	**MLP** R^2**/RMSE**	**LR** R^2**/RMSE**
ECPF4	0.686/1.136	0.655 / 1.1916	0.567 / 1.334
CVAE-None	0.568/1.334	0.557/1.349	0.452/1.502

CVAE-MolLogP	0.772/0.966	0.764/0.984	0.760/0.994
PVAE-None	0.745/1.022	0.668/1.165	0.641/1.212
PVAE-MolLogP	0.796/0.913	0.770/0.971	0.753/1.005

(b)

	**1D ResNet** $\mathbf{R^2}$**/RMSE**	**MLP** $\mathbf{R^2}$**/RMSE**	**LR** $\mathbf{R^2}$**/RMSE**
ECPF4	0.596/0.772	0.536 /0.827	0.426 / 0.920
CVAE-None	0.313/ 1.007	0.185/ 1.097	0.159/ 1.115
CVAE-MolLogP	0.397/ 0.943	0.292/ 1.023	0.276/ 1.034
PVAE-None	0.434/ 0.913	0.242/ 1.057	0.207/ 1.081
PVAE-MolLogP	0.520/ 0.840	0.319 / 1.001	0.296 / 1.018

(c)

	**1D ResNet** **Accuracy/F1**	**MLP** **Accuracy/F1**	**LR** **Accuracy/F1**
ECPF4	0.897/0.938	0.896 / 0.939	0.890 /0.934
CVAE-None	0.842/0.906	0.843/0.907	0.838/0.906
CVAE-MolLogP	0.865/0.921	0.862/0.917	0.867/0.922
PVAE-None	0.887/0.932	0.874/0.927	0.877/0.929
PVAE-MolLogP	0.888/0.934	0.876/0.928	0.880/0.931

Fig. S2 Clusters of the molecules from logS dataset in the latent space of a PVAE jointly pre-trained with a MolLogP predictor. Clusters are obtained using K-Means algorithm with varying number of clusters. In all cases there is one outlier cluster, without which there is a correlation between RMSE error of logS prediction and KL divergence between the cluster and the prior distribution

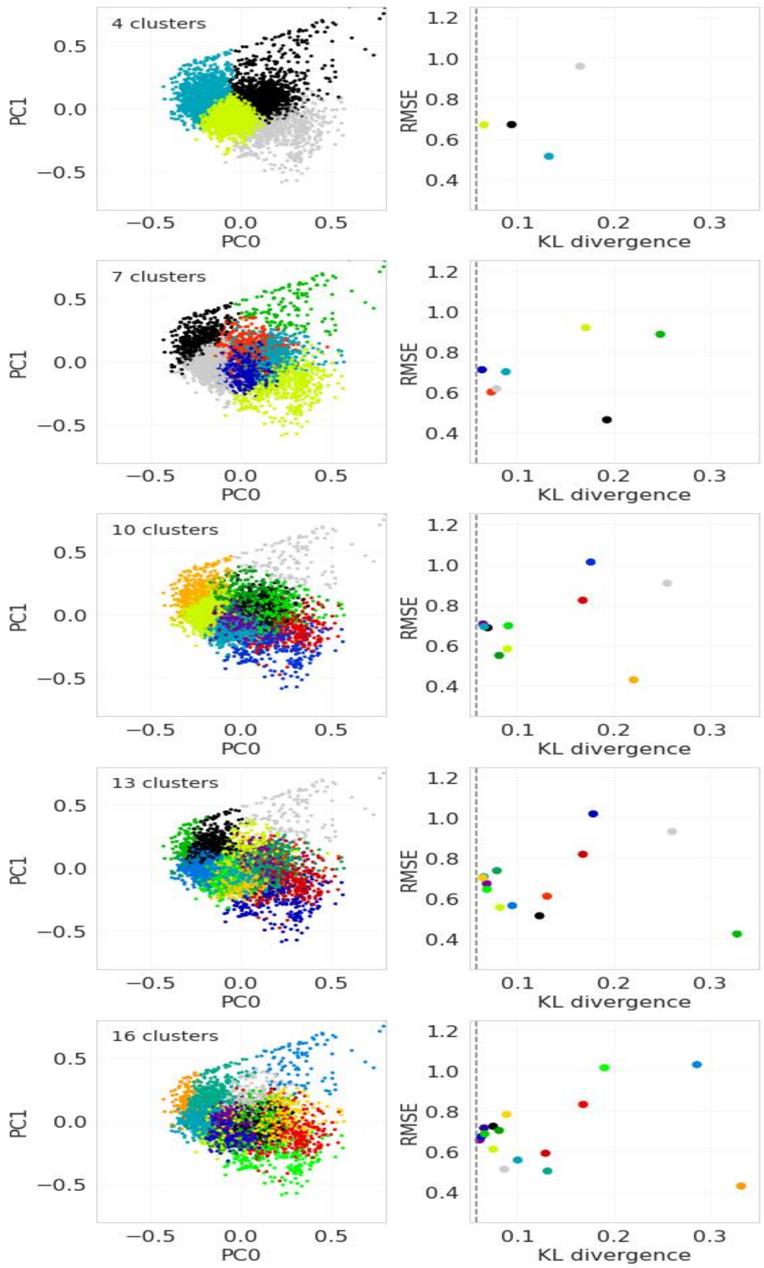

.
